# A Practical Approach to Intracranial Dural Arteriovenous Fistulas: Pathogenesis, Classification and Management

**DOI:** 10.3390/jcm14196895

**Published:** 2025-09-29

**Authors:** Karol Maciejewski, Miłosz Pinkiewicz, Bartosz Mruk, Daniel Knap, Artur Zaczyński, Jerzy Walecki, Michał Zawadzki

**Affiliations:** 1 Department of Radiology Warszawa, The National Medical Institute of the Ministry of the Interior and Administration, 02-507 Warsaw, Poland; 2 Department of Neurosurgery, The National Medical Institute of the Ministry of the Interior and Administration, 02-507 Warsaw, Poland; 3 Division of Interventional Neuroradiology, Department of Radiology, The National Medical Institute of the Ministry of the Interior and Administration, 02-507 Warsaw, Poland

**Keywords:** dural arteriovenous fistula, cerebrovascular malformation, radiosurgery, microsurgical resection, endovascular embolization, transvenous approach, transarterial approach

## Abstract

Dural arteriovenous fistulas (dAVFs) are a heterogeneous group of intracranial vascular anomalies characterized by abnormal arteriovenous shunting within the dura mater. While they are often considered acquired lesions—associated with trauma, surgery, venous sinus stenosis, or thrombosis—their precise etiology remains unclear in many cases. The clinical presentation of dAVFs varies widely depending on location and venous drainage patterns. Benign forms may manifest as pulsatile tinnitus or headache, whereas lesions with retrograde venous drainage and cortical venous reflux are considered aggressive and carry a heightened risk of hemorrhage and progressive neurological decline. Multiple classification systems, primarily based on angioarchitecture and venous outflow characteristics, have been developed to stratify risk and guide treatment strategies, as these features largely determine the natural history and clinical course of dAVFs. Endovascular embolization, microsurgical disconnection, and stereotactic radiosurgery (SRS) represent the mainstays of treatment, aiming to prevent hemorrhage or rebleeding and to alleviate symptoms related to venous congestion. Over the past two decades, advances in endovascular techniques have driven a paradigm shift in management, positioning embolization as the first-line therapy for most dAVFs. This review begins with a comprehensive overview of dAVF pathogenesis, classification systems, and angioarchitecture. It then focuses on the endovascular management of dAVFs, offering a detailed appraisal of current and emerging techniques, key technical considerations, and lesion-specific treatment strategies. Finally, we discuss the role of microsurgery and SRS.

## 1. Introduction

Intracranial dural arteriovenous fistulas (dAVFs) are vascular anomalies characterized by aberrant arteriovenous shunting confined to the dura mater, bypassing the normal capillary network [1]. Arterial supply most commonly arises from branches of the external carotid artery (ECA), but may also involve tentorial branches of the internal carotid artery (ICA), meningeal branches of the vertebral artery (VA), and, in complex lesions, pial branches of the cerebral circulation. The venous drainage of dAVFs may proceed anterogradely into dural sinuses or meningeal veins, or be redirected retrogradely into cortical or leptomeningeal veins as a result of elevated venous pressure within the fistulous circuit [2].

The pathogenesis of dAVFs remains incompletely understood, with the majority of cases arising without an identifiable inciting event. Nevertheless, accumulating evidence implicates acquired dural venous sinus thrombosis as a key initiating factor, precipitating venous congestion and intracranial hypertension that promote the development of pathological arteriovenous shunts. It is further hypothesized that chronic venous hypertension induces regional hypoperfusion, triggering angiogenic signaling cascades that contribute to the formation and progression of dAVFs [3].

DAVFs are rare, accounting for around 10% to 15% of all intracranial arteriovenous malformations, 6% of supratentorial, and 35% of infratentorial vascular malformations [4]. In terms of prevalence, dAVFs occur at a rate of approximately 0.15 cases per 100,000 individuals per year and are most frequently diagnosed in patients between 40 and 60 years of age [5,6,7,8,9]. Men are more likely to develop aggressive or complex dAVFs, which are associated with a higher risk of neurological morbidity and worse clinical outcomes [10]. The clinical presentation of dAVFs is highly variable and largely determined by altered venous flow dynamics and the anatomical location of the fistula. Symptoms range from pulsatile tinnitus, bruit, headaches, visual disturbances, alterations in mental status, seizures, myelopathy, cranial nerve palsies, and motor or sensory deficits to intracranial hemorrhage (ICH) [1].

Features such as cortical venous reflux (CVR), Galenic drainage, venous stenosis, and venous congestion are generally associated with an aggressive clinical course, including hemorrhage, non-hemorrhagic neurological deficits (NHNDs), and death. Reported annual hemorrhage rates in patients with CVR fall within the range of 8% to 13% [7,11]. Among patients presenting with hemorrhage, the risk of early rebleeding can be as high as 35% within the first two weeks following the initial ictus [12]. Conversely, patients with dAVFs without CVR are generally considered to have a benign clinical course, with an estimated annual hemorrhage risk approaching zero and a conversion rate to higher-grade lesions of approximately 1–2% during long-term follow-up [13,14].

Given the strong correlation between the natural history of dAVFs and their venous drainage patterns, multiple classification systems have been developed to stratify the risk of hemorrhage and neurological deterioration based on angioarchitectural features. Disease progression can vary considerably—some fistulas evolve insidiously over months or years, while others demonstrate rapid clinical deterioration. Accurate assessment of specific angioarchitectural characteristics, including lesion location, the presence of CVR, venous ectasia, and arterial supply patterns, is essential for evaluating the risk of aggressive behavior and selecting the most appropriate management strategy.

Endovascular embolization, microsurgical ligation, and stereotactic radiosurgery (SRS) are the primary treatment modalities for preventing hemorrhage or rehemorrhage and alleviating venous congestion-related neurological symptoms in patients with aggressive dAVFs [1]. In contrast, benign dAVFs are typically managed conservatively, as the risks of pursuing complete fistula occlusion often outweigh the very low risk of ICH [14]. Nevertheless, close clinical and radiographic follow-up is recommended due to the small but present risk of developing CVR [14]. Treatment is generally reserved for highly functionally limiting symptoms, with the primary objective being symptom relief rather than angiographic cure [1]. However, symptoms may persist following partial embolization, and many patients ultimately require further treatment to achieve complete obliteration and symptom resolution.

Owing to significant advancements in catheter technology and embolic agents, endovascular embolization has emerged as the primary treatment modality for dAVFs, demonstrating high efficacy in both symptom resolution and fistula occlusion, with reported complete occlusion (CO) rates ranging from 70% to 90% [15,16]. However, recurrences after initial angiographic cure are well documented, with long-term studies reporting rates between 9.5% and 14.3%, and these occur more frequently following endovascular than microsurgical obliteration. Consequently, microsurgical intervention remains a valuable alternative, either as a standalone treatment or in combination with embolization, particularly when endovascular options are unfeasible or have failed to provide a definitive cure [17,18]. For patients with complex dAVFs who are unlikely to achieve CO with embolization alone and are not optimal surgical candidates, SRS provides a minimally invasive option with a favorable safety profile and low complication rates [19].

This review begins by examining the pathogenesis of dAVFs, outlining current classification systems, and providing a comprehensive overview of the angioarchitectural features that define each dAVF subtype. The second part of the review focuses on endovascular management, offering a detailed assessment of both established and emerging techniques. We discuss in detail lesion-specific considerations that critically inform therapeutic decision-making and influence patient outcomes. Lastly, we address how microsurgery and SRS serve as complementary treatment options.

## 2. Methodological Approach

A targeted literature search was performed using the PubMed and Cochrane databases to identify relevant publications on dAVFs. Search terms included “dural arteriovenous fistula,” “cerebrovascular malformation,” “radiosurgery,” “microsurgical resection,” “endovascular embolization,” “transvenous approach,” “transarterial approach”, “balloon-assisted embolisation”, “pressure-cooker technique”, “reverse pressure cooker technique”, and “liquid embolic agents”. Articles published in English were considered, with no restriction on publication date. We have included studies on epidemiology, pathophysiology, classification systems, angioarchitecture, and treatment of dAVFs.

References of selected articles were also screened to identify additional relevant studies. Our focus was primarily on various aspects of endovascular management, but studies discussing microsurgery and radiosurgery were also included. The aim was to provide a comprehensive, up-to-date synthesis of current knowledge, highlighting key clinical considerations and emerging trends in the management of intracranial dAVFs.

## 3. Results

Our literature search identified 2756 articles, of which 1532 remained after removing duplicates. After screening titles and abstracts, 902 articles were assessed for relevance. Following full-text review and application of inclusion and exclusion criteria, 146 publications were ultimately selected for synthesis.

### 3.1. Pathogenesis and Pathophysiology

The pathological mechanisms underlying dAVF formation have yet to be fully understood. Only a minority of cases are considered congenital, arising from early embryological vascular maldevelopment, likely due to the failure of the primitive vascular plexus to form a normal capillary network, resulting in persistent arteriovenous channels [2,3]. Evidence for a genetic predisposition to DAVFs is extremely limited, with only a single case report describing two first-degree relatives, both harboring prothrombin mutations [20].

The majority of dAVFs, however, are idiopathic, with a subset developing secondary to local trauma, infection, neoplasia, or surgical interventions involving the dura. In both forms, dural venous sinus thrombosis appears to be a unifying pathogenic factor [21].

Accumulating evidence supports a mechanistic model in which venous hypertension and thrombosis trigger a cascade of inflammatory and angiogenic responses, ultimately promoting the development of arteriovenous shunts within the dura mater [22]. The observed association between dAVFs and hereditary thrombophilic conditions—such as Factor V Leiden mutation and protein C or S deficiencies—strongly supports this hypothesis [3,14]. Similarly, recent studies have reported a significant link between the MMP-2-1306 C allele and sinus thrombosis in dAVF patients, likely mediated by enhanced extracellular matrix degradation and increased platelet aggregation. An imbalance between matrix metalloproteinase-2 (MMP-2) and its endogenous inhibitor TIMP-2 may promote prothrombotic venous remodeling, predisposing to CVR and more aggressive angioarchitectural patterns [23].

Venous thrombosis—identified in up to 30% of patients with dAVFs—leads to progressive sinus stenosis or occlusion, resulting in elevated venous pressure and the initiation of compensatory arteriovenous shunting [8]. A pathological feedback loop ensues: arterialized blood entering the venous system exacerbates congestion and turbulence, promoting further thrombosis and sustaining venous hypertension. This elevated pressure induces regional hypoxia, which stimulates angiogenic pathways mediated by factors such as VEGF and HIF-1α, facilitating fistula formation and vascular remodeling [24]. Nrf2, a transcription factor involved in oxidative stress regulation, has also been implicated in this response. If venous pressure remains unresolved, persistent endothelial injury and aberrant vessel proliferation may reinforce the cycle [25]. Supporting this model, Miyachi et al. demonstrated that inflammatory changes at the penetration sites of emissary veins can induce neovascularization and microvascular arteriovenous channel formation. As these shunts mature, they may recruit arterial supply from distant territories, evolving into aggressive dAVFs with impaired venous drainage and CVR. This tissue-level mechanism corroborates the broader pathophysiological framework and may account for both sinus-type and non-sinus-type lesions [21]. Clinical observations further validate this hemodynamic model. In select cases, endovascular recanalization of stenosed venous sinuses via balloon angioplasty or stenting has led to spontaneous fistula closure, likely through normalization of local venous pressures and restoration of physiological venous drainage, thereby eliminating the pressure gradient that sustains arteriovenous shunting [26]. A clear understanding of these mechanisms is pivotal for advancing our knowledge of dAVF pathogenesis and informing both current therapeutic approaches and future interventions.

Pharmacologic agents have also been suggested as potential triggers for dAVFs. A decade-long study utilizing pharmacovigilance analysis of the FAERS database systematically assessed the risk of drug-induced dAVF, identifying tamoxifen, methylprednisolone, betamethasone, prednisone, rebif, ustekinumab, natalizumab, baclofen, dabigatran etexilate, and bupivacaine as having the potential to induce these lesions. Among these, tamoxifen presents the most significant risk, possibly due to its role in increasing thrombosis and regulating angiogenesis [27]. Future research should prioritize elucidating the underlying mechanisms and risk factors, such as thrombosis, contributing to drug-induced dAVFs, to inform preventive strategies and optimize patient care.

The meningeal immune system (MIS)—comprising the meninges, meningeal vasculature, nerve branches, and resident neuro-immune cells—has attracted increasing attention in understanding dAVF pathogenesis [28]. It is hypothesized that abnormal vascular shunting in dAVFs may represent a compensatory response mediated by MIS surveillance and regulation. Furthermore, given the role of meningeal lymphatic vessels (MLVs) in cerebrovascular repair and remodeling, it is plausible that dAVF formation is closely linked to meningeal lymphatic activity, potentially representing an imperfect byproduct of the repair process [28]. Further studies are necessary to clarify the precise role of the MIS and MLVs in dAVF formation.

Although spontaneous regression of dAVFs has been reported, the underlying mechanisms remain poorly understood. Proposed explanations include thrombosis of the fistula, turbulent flow disruption, trauma-induced vessel remodeling, or compression of feeding arteries by adjacent hematomas [29]. Moreover, the development of multiple dAVFs in some patients may reflect cycles of thrombotic occlusion and recanalization, further reinforcing the role of venous hypertension as a central pathogenic driver [30].

Up to 21% of patients with DAVFs have concomitant aneurysms, with roughly one-third on feeding arteries and two-thirds in remote locations [31]. Aneurysms on feeding arteries may result from flow-related stress, endovascular treatment (EVT), or the initial inciting event, such as trauma [31]. Remote aneurysms, however, suggest a potential genetic predisposition or intrinsic vulnerability in cerebrovascular architecture that may facilitate aneurysm formation alongside DAVFs [31]. Future studies should explore which DAVF angioarchitectural features predispose to feeding artery aneurysms and examine how hemodynamic changes following EVT might contribute to de novo aneurysm development. Finally, integrating genetic analysis with detailed vascular phenotyping is warranted to clarify whether a heritable component contributes to lesion formation.

Continued investigation into the pathogenesis of dAVFs is essential. A more comprehensive understanding of the thrombotic, inflammatory, and angiogenic mechanisms involved may inform the development of pharmacological strategies aimed at preventing lesion progression or facilitating spontaneous regression. Whether environmental factors contribute to DAVF formation is unknown, warranting further research to identify potential risk factors.

### 3.2. Angioarchitecture

The arterial supply to dAVFs is notably diverse, reflecting both the complexity of intracranial vascular anatomy and the dynamic nature of pathological vascular remodeling. DAVFs can be supplied by arteries arising through normal anatomical routes, persistent embryologic connections, or newly developed collateral pathways that form in response to local hemodynamic changes. Classical dural supply arises from extradural arteries, most commonly the meningeal branches of the ECA and ICA, with the middle meningeal artery (MMA) and occipital artery (OA) being the most frequently involved. In addition, some fistulas are supplied by dilated pre-existing or congenital dural branches of intradural pial arteries, such as the artery of Davidoff and Schechter. More rarely, a newly developed ‘pure’ pial supply may emerge from tortuous distal branches of cerebral arteries outside of known physiologic or embryologic connections to the dura [32]. Pial arterial involvement, present in over 10% of cases and particularly common in tentorial dAVFs (T-dAVFs), is associated with more aggressive clinical behavior and a higher risk of hemorrhage [33,34].

Venous drainage may occur through dural venous sinuses, cortical veins, or perimedullary veins. The pattern of venous outflow is the principal determinant of dAVF classification and strongly correlates with symptom severity and hemorrhagic risk [35]. These angioarchitectural features—arterial feeders and venous drainage routes—are detailed for selected dAVF subtypes in Table 1 [36,37,38,39,40,41,42]. Shapiro et al. introduced a refined angioarchitectural model for dAVFs involving the transverse and sigmoid sinuses, centered on the concept of a “common arterial collector.” This refers to a segment within the sinus wall where multiple arterial feeders converge before draining into the lumen of the dural venous sinus through a discrete fistulous connection. This anatomical insight supports superselective transvenous embolization targeted at the collector, enabling effective fistula closure while minimizing collateral damage and preserving venous outflow [43].

A deep understanding of the anatomical location of dAVFs is essential, as each site poses distinct diagnostic and therapeutic challenges. The most clinically relevant locations include the cavernous sinus, transverse and sigmoid sinuses, superior sagittal sinus, anterior cranial fossa, and tentorial region [10]. However, dAVFs may also arise in less common sites such as the posterior condylar canal or the hypoglossal canal, among others [44,45]. Notably, the distribution of dAVFs across anatomical locations varies considerably among published series [10,46].

#### 3.2.1. Transverse Sigmoid Sinus Dural Arteriovenous Fistulas

Transverse-sigmoid sinus dAVFs (TS-dAVFs) represent the most prevalent subtype of intracranial dAVFs, most commonly localized at the transverse–sigmoid sinus junction [37,47]. Their clinical presentation is heterogeneous, encompassing pulsatile tinnitus, insomnia, headache, cranial bruit, visual deterioration, seizures, and, in some cases, altered mental status. Among these, pulsatile tinnitus and headache are the most frequently reported symptoms [47].

The vascular supply to TS-dAVFs typically arises from two distinct arterial systems. The first involves transosseous feeders, characterized by tortuous, often multiple arterial branches converging from a single trunk—most commonly from transosseous branches of the OA and, less frequently, the superficial temporal artery. The second pattern comprises meningeal feeders, predominantly the petrosquamous branch of the MMA, which is frequently recruited in fistulas involving the transverse–sigmoid region. Unlike the transosseous route, meningeal arteries enter the cranium through the skull base, offering a more favorable and linear trajectory for microcatheter navigation and embolic delivery [48].

The angioarchitecture of TS-dAVFs frequently includes shunted pouches (SPs) defined as tubular or elliptic vascular structures separated from the main lumen of the dural sinus, into which multiple arterial feeders converge and from which the arteriovenous shunt drains into the transverse–sigmoid sinus [49]. Two distinct morphologic types of SPs have been described: (1) a “septation,” representing a compartmentalized segment within the dural sinus itself; and (2) an “accessory sinus,” a venous channel located outside the sinus lumen [50]. SPs are most commonly situated at the transverse–sigmoid junction but may also extend into adjacent segments, including the transverse sinus, proximal sigmoid sinus, or torcular region. The exact topographic location of an SP critically influences the venous drainage pattern—particularly the presence or absence of CVR—and directly impacts the feasibility and safety of both arterial and transvenous access routes. These pouches are typically supplied by transosseous branches of the OA and the petrosquamous segment of the MMA [49].

#### 3.2.2. Superior Sagittal Sinus Dural Arteriovenous Fistulas

Although superior sagittal sinus dAVFs (SSS-dAVFs) are relatively rare, accounting for approximately 5–12% of all intracranial dural AVFs, they are complex lesions due to their frequent bilateral arterial supply and involvement of extensive, often eloquent territories. SSS-dAVFs can be broadly classified into two distinct angioarchitecture subtypes: the first involves direct arterialization of the superior sagittal sinus itself, while the second comprises parasagittal arteriovenous shunts that drain into cortical veins adjacent to the sinus, with retrograde CVR and only secondary involvement of the sinus [51,52].

In a comprehensive review of 51 patients with SSS-dAVFs undergoing EVT, the MMA was implicated in all cases, with bilateral MMA involvement reported in 78.4% of patients. Other commonly recruited arterial feeders included the superficial temporal artery (62.7%) and the OA (49.0%). Notably, pial arterial supply—often associated with higher-grade and more aggressive fistulas—was identified in 21.6% of cases. Venous outflow restriction, including stenosis or occlusion of the superior sagittal sinus, was observed in 37.3% of patients. Generally, aggressive lesions characterized by CVR and a heightened risk of hemorrhagic or neurological complications constitute the majority. According to the Borden classification, Type I lesions accounted for 7.8% of cases, Type II for 37.3%, and Type III for 54.9%, reflecting a predominance of high-grade lesions with CVR and increased risk of hemorrhagic or neurological complications [38].

#### 3.2.3. Cavernous Sinus Dural Arteriovenous Fistulas

Cavernous sinus dAVFs (CS-dAVFs) account for approximately 16% of all intracranial dAVFs. These lesions are most often unilateral, although bilateral involvement has been reported in 14.2% to 26% of cases [53]. The predominant clinical manifestations arise from venous hypertension within the ophthalmic venous system, leading to ocular symptoms such as chemosis, proptosis, and cranial nerve III, IV, or VI palsies. Less frequently, patients may present with headache, retro-orbital pain, pulsatile tinnitus, or reduced visual acuity [54]. Liu et al. in a study of 64 patients reported that for 11 patients the feeding artery was from ECA, while in the rest of the patients, the CS-dAVFs were supplied by both the ECA and ICA [55]. MMA, accessory meningeal artery, and ascending pharyngeal artery (APhA) are considered to be the most clinically significant branches of these arteries. Regarding venous drainage, the superior ophthalmic vein (SOV) is most commonly involved in patients with CS-dAVFs, whereas drainage through the inferior petrosal sinus (IPS) and cortical veins occurs less frequently [56].

#### 3.2.4. Parasellar Dural Arteriovenous Fistulas

In addition to the cavernous sinus, dAVFs may also arise in the parasellar region, where they often mimic the clinical features of CS-dAVFs, potentially leading to misdiagnosis. These lesions typically involve venous structures that communicate with the cavernous sinus, including the SOV, sphenoparietal sinus, superficial middle cerebral vein (SMCV), uncal vein, venous plexus of the foramen ovale (pterygoid venous plexus), IPS, basilar venous plexus, and superior petrosal sinus (SPS). Hiramatsu et al. divided parasellar AVFs, according to their anatomical relationship to the CS, into four groups: anterior group (orbit), anterolateral group (sphenoid wing), posteroinferior group (IPS and clivus), and posterior group (SPS and petrosal vein) [Table 2] [36].

#### 3.2.5. Tentorial Dural Arteriovenous Fistulas

T-dAVF are rare, accounting for 4–8.4% of all intracranial dAVF cases [57]. These lesions are located within the reflected (double-layer) dura of the tentorium and its dural attachments and are characterized by a complex angioarchitecture and aggressive clinical behavior. T-dAVFs almost invariably drain into leptomeningeal veins, corresponding to Cognard types III and IV, and are therefore associated with a high risk of ICH [58]. A meta-analysis of 377 dAVF patients revealed that 97% of T-dAVF cases resulted in hemorrhage or progressive focal neurological deficits, highlighting their particularly severe clinical impact [59].

The complex and variable anatomy, along with the diverse clinical behavior of tentorial dAVFs, prompted the development of specialized classification systems: Lawton’s emphasizes microsurgical anatomy and venous drainage to guide operative planning [60] [Table 3]; Picard’s is based on topographic anatomy and associated clinical syndromes [61] [Table 4] and Lasjaunias’ focuses on angiographic features and deep venous outflow [62] [Table 5].

T-dAVFs receive arterial supply from the external and internal carotid systems, as well as from the vertebrobasilar circulation, with the specific arterial contributors varying according to the fistula’s topographic location within the tentorial dura. Branches of the ECA, particularly the MMA and the OA, are commonly involved. The MMA typically supplies fistulas situated more anteriorly or laterally along the tentorium, whereas the OA is more frequently implicated in fistulas located along the posterior tentorium or near the torcular Herophili. In high-flow or extensive lesions, additional feeders such as the accessory meningeal artery and the APhA may be recruited [63].

From the internal carotid system, the most significant contributor is the marginal tentorial artery, especially in lesions near the petroclival junction or the anteromedial tentorium. In certain anatomical configurations, the inferolateral trunk may also supply the lesion, especially when it extends toward the petrous apex or adjacent dural structures [63,64].

The vertebrobasilar system also provides important arterial feeders, particularly in medial and posteriorly located T-dAVFs. The medial tentorial branch of the superior cerebellar artery is a key contributor to midline lesions, especially those near the straight sinus or falx cerebelli. The posterior meningeal artery, typically arising from the VA, is another frequent source in fistulas located along the inferior surface of the tentorium [65]. Of particular clinical relevance is the artery of Davidoff and Schechter, which can supply medial or high-grade T-dAVFs. Its involvement is especially significant in lesions that drain into deep venous structures such as the vein of Galen or straight sinus, where the risk of hemorrhage is substantial due to the high-flow and retrograde venous drainage patterns [40].

A thorough understanding of this complex arterial anatomy is critical for safe and effective treatment. Many of these arterial feeders are in close proximity to cranial nerves, or supply the brainstem, thus elevating the risk of iatrogenic injury during treatment. For this reason, meticulous angiographic assessment is essential to delineate the vascular architecture and to guide the choice of therapeutic strategy.

#### 3.2.6. Anterior Cranial Fossa Dural Arteriovenous Fistulas

Anterior cranial fossa dAVFs (ACF-dAVF), also known as ethmoidal dAVFs, represent approximately 10% of all intracranial dAVFs. They are typically located at the level of the cribriform plate—either centrally or slightly off-midline—within the lateral epidural compartment adjacent to the cribriform plate and the orbital roof. Owing to the absence of a dural venous sinus in the anterior cranial fossa, ACF-dAVFs drain directly into pial veins, most commonly via the olfactory vein into the basal vein of Rosenthal, or through ascending frontal cortical veins into the superior sagittal sinus. This pattern of direct cortical or deep venous drainage, without an intervening sinus, defines their classification as Borden type III and Cognard type III or IV fistulas [66]. The reliance on cortical veins for venous outflow may lead to venous engorgement and increased intravascular pressure, contributing to the lesions’ aggressive clinical course. Reported rates of intracranial hemorrhage at presentation range from 62% to 91%, underscoring the need for prompt intervention irrespective of symptomatology. The arterial supply to ACF-dAVFs is complex and highly variable, typically involving small terminal branches arising from both the ICA and ECA systems. The anterior and posterior ethmoidal arteries are the most consistent arterial feeders [42,67]. Additional supply may arise from the frontal branch of the MMA (20% of cases), and from the septal branch of the sphenopalatine artery, which forms anastomoses with the ethmoidal arteries (20–60% of cases) [42,68,69].

#### 3.2.7. Anterior Condylar Confluence Dural Arteriovenous Fistulas

Anterior Condylar Confluence dAVFs (ACC-dAVFs) are rare, comprising only 1.8–3.6% of intracranial dAVFs, but are notorious for their complex venous anatomy and variable drainage patterns. The craniocervical junction’s intricate architecture and overlapping venous structures have made consistent nomenclature difficult [45,70]. As a result, these fistulas are variably described as involving the hypoglossal canal, anterior condylar vein, inferior petrosal sinus, marginal sinus, jugular foramen, or foramen magnum [70].

The arterial supply of ACC dAVFs typically arises from ECA branches, most consistently the APhA. Additional feeders may include the OA, MMA, and posterior auricular artery (PAA). In some cases, contributions from the ICA—particularly the meningohypophyseal trunk (MHT)—or branches of the VA may also be present [70,71,72]. The clinical presentation of ACC-dAVFs is highly variable and dictated by venous drainage patterns. Anterograde flow into the IJV or vertebral venous plexus (VVP) typically causes pulsatile tinnitus, whereas retrograde drainage into the IPS or CS may lead to orbital symptoms (e.g., diplopia, proptosis, chemosis), mimicking CS-dAVFs [73,74].

Less commonly, congestive myelopathy may occur due to venous hypertension of the anterior spinal vein, presenting with limb weakness, urinary dysfunction, and cord edema [75]. The close proximity of the ACC venous plexus to the hypoglossal canal predisposes the hypoglossal nerve to compression or ischemia, with palsy reported in ~11.7% of cases. Although rare (~5%), ICH remains a serious complication, particularly in the setting of perimedullary drainage [76].

### 3.3. Classifications

The earliest attempt to classify dAVFs based on venous drainage patterns and clinical presentation was introduced by Djindjian, Merland, and Theron. Their system defined four subtypes according to drainage patterns and the presence of reflux or venous varices, establishing the fundamental principle that CVR is the main predictor of aggressive clinical behavior [77]. Although clinically intuitive and widely used at the time, this system lacks the granularity required for precise risk stratification and detailed angioarchitectural assessment [78].

In current clinical practice, the most widely adopted classification systems are those of Borden and Cognard. The Borden classification stratifies dAVFs based on the site of venous drainage and the presence or absence of CVR. While it effectively distinguishes high-risk lesions, it relies on a binary interpretation of CVR and does not consider more nuanced venous outflow dynamics [79]. The Cognard classification builds upon this by incorporating additional features such as the direction of flow within the dural sinus, the presence of venous ectasia, and the specific architecture of the venous drainage. This classification framework is more comprehensive, reflecting the substantial heterogeneity observed among type II DAVFs and incorporating type V DAVFs, in which spinal venous drainage underlies the development of myelopathy. This classification framework is more comprehensive, reflecting the substantial heterogeneity observed among type II DAVFs and incorporating type V DAVFs, in which spinal venous drainage underlies the development of myelopathy. Despite its widespread use, the Cognard classification system exhibits certain limitations. First, its categories may lack sufficient granularity to accurately stratify risk in all cases [14], particularly for lesions with mixed or atypical venous drainage patterns. Second, the system does not account for the dynamic nature of dAVFs, which can evolve over time, potentially altering their risk profile after initial imaging. Finally, accurate classification relies heavily on expert interpretation of complex angiographic findings, introducing potential inter-observer variability that may affect both diagnosis and subsequent management decisions. Collectively, these limitations underscore the need for continued refinement of classification frameworks to enhance predictive accuracy and clinical utility.

In contrast to these venous drainage–oriented classifications, the Barrow system focuses on arterial angioarchitecture and is applicable exclusively to carotid–cavernous fistulas (CCFs) [80]. It differentiates between direct fistulas, involving high-flow shunts from the intracavernous ICA (type A), and indirect fistulas, in which the shunt is supplied by dural branches of the ICA or ECA (types B, C, and D). Importantly, only the indirect variants are considered true dAVFs, as the arteriovenous shunting occurs within the dural walls of the cavernous sinus. This anatomical distinction has led to increasing support for replacing the term “indirect CCF” with “parasellar dAVF,” a nomenclature that more accurately reflects the lesion’s dural origin and aligns with the classification of other intracranial dAVFs [3]. However, the Barrow system is of limited value from a therapeutic standpoint as it does not incorporate venous drainage characteristics—widely recognized as the primary determinant of clinical behavior, symptom severity, and treatment planning. It also lacks prognostic value, given that it does not account for the presence or absence of CVR, a key predictor of aggressive clinical course [80]. To address this limitation, Thomas et al. proposed a classification for CCFs based on venous drainage direction, distinguishing between anterior and posterior pathways. This framework has demonstrated strong correlation with symptomatology and is increasingly used to guide intervention [81,82]. Other classification systems have focused specifically on CS-dAVFs. Suh et al. introduced a model based on angioarchitectural and hemodynamic behavior, dividing CS-dAVFs into proliferative, restrictive, and late-restrictive subtypes. This approach considers both morphological complexity and drainage patterns, allowing for a more functional assessment of lesion severity and clinical progression [83]. Similarly, Wenderoth and colleagues developed a classification based on the patency of the IPS, identifying variants with preserved, partially obstructed, or absent IPS outflow. This system has proven particularly helpful in procedural planning for transvenous embolization [84].

The Zipfel classification was proposed to address some of the limitations of existing dAVF grading systems, focusing on the clinical relevance of cortical venous drainage (CVD) and symptomatic status rather than solely on venous anatomy. The underlying concept is that dAVFs with cortical venous involvement pose a higher risk for hemorrhage and neurological deficits, and incorporating symptomatic information can better guide clinical decision-making. One key advantage of this approach is its direct link between angiographic features and clinical risk, potentially improving prioritization for intervention. Additionally, by simplifying categories, it offers a more intuitive framework for clinicians, reducing ambiguity in interpretation [78] [Table 6]. The Geibprasert classification provides an embryology-based framework for understanding intracranial DAVFs, grouping lesions according to the developmental origin of the dural venous sinuses into ventral, dorsal, and lateral compartments. These categories have been associated with distinct clinical patterns in observational studies, with ventral lesions generally reported as less aggressive and lateral lesions more likely to exhibit high-risk features [85]. The main advantage of this system is its mechanistic perspective, linking differential aggressiveness to developmental patterns rather than relying solely on venous drainage. However, it does not explicitly account for dynamic lesion evolution or patient-specific symptomatology, restricting its use for immediate risk stratification in individual patients.

While these classification systems provide essential frameworks to understand dAVF anatomy, angioarchitecture, and natural history, they do not predict treatment outcomes such as the likelihood of fistula obliteration. To address this gap, the VEBAS score (range 0–12) was developed as a practical grading system that estimates the probability of complete obliteration and favorable outcomes following dAVF treatment, thereby aiding patient counseling and setting realistic treatment expectations. Internally validated using the Consortium for Dural Arteriovenous Fistula Outcomes Research (CONDOR) database, the VEBAS score incorporates patients’ baseline characteristics (age and prior cranial surgery), dAVF angiographic features (venous stenosis and arterial feeders), and the Borden classification. In the validation cohort, patients with scores of 0–3 had a predicted zero occlusion rate, whereas those scoring 8–12 had occlusion rates between 72% and 89%. Each additional point in the VEBAS score corresponded to a significant increase in the likelihood of complete obliteration and favorable outcome [86].

**Table 6:** Chosen classification of dAVFs (Reused from Zhang et al.) [87].

Djindjian-Merland’s	Borden’s	Cognard’s	Zipfel’s
I, drainage into a sinus	I, venous drainage directly into the dural venous sinus or the meningeal vein	I, venous drainage into the dural venous sinus with antegrade flow	I, dAVFs drain directly into dural venous sinuses; spinal extra-dural AVM without perimedullary vein reflux
		IIa, venous drainage into the dural venous sinus with retrograde flow	II, dAVFs drain into dural sinuses but also have retrograde drainage into ophthalmic and bridging veins; spinal extra-dural AVM with perimedullary vein reflux
II, sinus drainage with reflux into the cerebral veins	II, with cortical vein reflux	IIb, antegrade dural venous drainage with cortical venous reflux	
		IIa+b, retrograde dural venous drainage with cortical venous reflux	
III, drainage solely into cortical veins	III, cortical vein drainage	III, venous drainage directly into cortical veins	III, dAVFs drain into pial veins and do not have dural sinus drainage; spinal dAVF drained with perimedullary vein only
IV, with supra or infra tentorial venous lake		IV, type III with venous ectasias of the draining subarachnoid veins	
-	-	V, spinal perimedullary vein drainage	II or III, cranial or spinal dAVF

### 3.4. Endovascular Treatment

The therapeutic paradigm for dAVFs has progressively shifted from open surgical interventions toward endovascular embolization as the predominant first-line treatment, driven primarily by advances in microcatheter technology and the development of effective liquid embolic agents (LEAs). These include n-butyl-2-cyanoacrylate (n-BCA, Trufill, DePuy Synthes), Onyx (ev3 Endovascular), Squid (Emboflu), precipitating hydrophobic injectable liquid (PHIL; MicroVention) [Table 7], and detachable microcoils, which have largely supplanted earlier agents such as detachable balloons, polyvinyl alcohol particles, silk sutures, and microspheres [1]. This evolution has been further supported by accumulating clinical evidence demonstrating the safety and efficacy of endovascular embolization as a first-line treatment in carefully selected patients [10,12,46,47,84,85].

EVT for dAVFs can be performed either via transvenous embolization (TVE) or transarterial embolization (TAE). TVE involves retrograde catheterization of the affected dural sinus or cortical vein, followed by deposition of coils and/or LEAs adjacent to the shunt. The main challenge is achieving gradual, controlled obliteration of the fistula without diverting arterialized blood into cortical veins. However, occlusion of a dural sinus during TVE carries a risk of venous infarction or hemorrhage, which limits its applicability [88,89]. TAE, in contrast, involves superselective distal catheterization of arterial feeders with delivery of an LEA [88]. This sinus-preserving approach is often preferred in low-grade fistulas, as it allows maintenance of the normal venous drainage pattern.

Ultimately, the choice of endovascular route is tailored to the fistula’s location, venous drainage pattern, and the feasibility of selective arterial or venous catheterization.

The cornerstone of EVT is embolization of the fistulous connection and its venous components while avoiding adverse hemodynamic effects. A thorough understanding of the fistula’s arterial and venous anatomy is essential before treatment, as incomplete or inappropriate embolization can cause adverse hemodynamic remodeling, including pressure redistribution, venous congestion, and recruitment of new arteriovenous connections [1]. This risk is particularly high in lesions with preexisting sinus occlusion, where redirected flow may exacerbate cortical venous hypertension and increase hemorrhage risk [3]. Nonetheless, partial embolization can be justified in certain clinical scenarios. In high-grade dAVFs with CVR where complete occlusion is not feasible, partial embolization aimed at disconnecting the CVD can reduce the risk of hemorrhage or rehemorrhage. Similarly, partial embolization is commonly employed in low-grade dAVFs to alleviate functionally limiting symptoms. However, symptoms may persist following partial embolization, with patients ultimately requiring staged or combined treatments to achieve complete obliteration and symptom resolution [89]. Although partial treatment carries a risk of arterial occlusion and retrograde venous flow, which may theoretically promote CVR development and progression to higher-grade dAVFs, recent evidence refutes this hypothesis [90]. Analysis of data from the multi-institutional CONDOR database, the largest registry of cranial dAVF patient data in the world, encompassing 337 patients with type I dAVFs, revealed no statistically significant increase in the risk of conversion from low- to high-grade lesions among partially treated patients compared to those managed conservatively or treated to angiographic cure [91]. The high-grade conversion rate was 2.2% for partially treated type I dAVFs versus 1.5% for conservatively managed lesions, aligning with prior reports showing infrequent conversion rates (0–8%) in this population [13,92].

#### 3.4.1. Transarterial Embolization

TAE is generally the preferred treatment for high-grade dAVFs with direct CVR, offering several advantages over the TVE. These include a reduced risk of flow redirection into alternate venous drainage pathways, preservation of functional venous sinuses, and a potentially lower risk of de novo dAVF formation secondary to venous hypertension. Additionally, TAE avoids complications associated with transvenous navigation, such as cranial nerve palsies (e.g., abducens nerve injury during catheterization of the IPS or SPS [1,93,94,95]. Achieving durable angiographic cure with endovascular therapy requires complete embolization of both the fistulous connection and the proximal draining vein. Even when angiographic flow cessation is initially observed following proximal occlusion, small-caliber feeders—often angiographically occult—may persist or be recruited over time, ultimately resulting in recanalization and lesion recurrence. This underscores the importance of meticulous technique and comprehensive assessment of all potential arterial contributors to ensure long-term treatment success.

Special care during TAE should be taken to selectively occlude only the proximal segment of the draining vein, as inadvertent embolization of the distal venous system may lead to progressive venous outflow obstruction, exacerbation of venous hypertension, and potentially venous infarction [88]. If venous ectasia or aneurysms are present along the draining vein, they constitute the most common source of bleeding. These should either have their outflow preserved or be occluded together with the dAVF’s proximal draining vein. Inadvertent migration of the embolic agent into the distal part of the draining vein may obstruct outflow and cause serious bleeding due to increased pressure within the venous aneurysm [94]. Aneurysms on the feeding artery require careful evaluation, as hemodynamic changes induced by dAVF embolization may increase the risk of rupture. Preemptive embolization of the aneurysm prior to EVT of the dAVF may help prevent subsequent rupture. Conversely, in selected cases where the aneurysm is small or deemed low-risk, it may be reasonable to first treat the dAVF and monitor the aneurysm, addressing it only if it enlarges or demonstrates high-risk features. Treatment strategy should be individualized based on aneurysm morphology, location, and overall hemodynamic context.

#### 3.4.2. Flow Control Techniques

Given that the success of TAE hinges on achieving a “wedged” microcatheter position that facilitates forward embolic flow while minimizing reflux, flow-arrest techniques have been developed to enhance safety and efficacy of EVT [1,95,96]. The pressure cooker technique (PCT) is a double-microcatheter strategy designed to enable more controlled and extensive embolization by preventing proximal reflux of the embolic agent and reducing the risk of non-target embolization, particularly in anatomically complex lesions [95,97]. This technique involves deploying a coil and/or glue plug via a proximal microcatheter to create a mechanical barrier within the feeding artery, followed by injection of an LEA through a second, more distal microcatheter positioned near the fistulous point. The resulting closed system facilitates safe, high-pressure antegrade filling of the fistulous pouch and its draining vein, improving the likelihood of complete occlusion in a single session.

Microballoon-assisted embolization has emerged as a technically simpler and potentially safer method to achieve durable flow arrest and controlled delivery of liquid embolic agents. The advent of double-lumen balloon catheters, which enable simultaneous temporary flow arrest and embolic injection via a single access, has further enhanced procedural efficiency, reducing occlusion times by half compared to single-lumen catheters and improving rates of immediate complete occlusion. While conventional DLBCs were limited in accessing small, tortuous vessels due to relatively large distal profiles and reduced flexibility, the Scepter Mini (MicroVention, Aliso Viejo, CA, USA)—featuring a semi-compliant 2.2 × 9 mm balloon and a 1.6 French distal outer diameter—has demonstrated significantly improved distal navigability, with successful use reported in vessels as small as 0.7 mm [98,99]. However, its low-profile design is not without limitations. The small balloon diameter may result in inadequate anchoring in larger vessels, allowing minimal proximal reflux of the embolic agent around the balloon. This may compromise flow arrest and embolization stability, particularly in high-flow lesions or larger arteries where full occlusion of the vessel is not achieved [98]. 

While flow-arrest techniques reduce reflux and enhance forward penetration of LEAs, elevated intraluminal pressure within the sealed segment may inadvertently propel the embolic agent deeper into the venous system than intended, especially in high-resistance shunts or compromised venous outflow. This highlights the importance of meticulous technique and detailed angioarchitectural evaluation when employing PCT or balloon-assisted embolization [96,100,101].

Both the PCT and microballoon-assisted embolization improve occlusion rates compared to conventional TAE. In a series of 68 dAVFs, complete occlusion was achieved in 72%, 79%, and 86% of cases treated with conventional TAE, PCT, and microballoon-assisted TAE, respectively [102]. However, PCT requires the use of multiple microcatheters to construct a proximal plug ahead of the most distal catheter used for LEA injection, making the technique inherently more technically demanding and time-consuming. In contrast, microballoon-assisted embolization obviates the need for a second microcatheter and additional embolic materials, thereby reducing the risk of complications such as glue reflux into unintended arterial territories, which may result in ischemia or cranial nerve palsy. The greater complexity of PCT is reflected in its higher complication rate (29%) compared with conventional TAE (17%) and microballoon-assisted TAE (7%), as well as its association with longer fluoroscopy times [102].

#### 3.4.3. Flow Diversion

Flow diversion was initially employed in the treatment of direct CCFs. Castãno et al. later reported two cases of indirect CCFs (Barrow Type B), a subtype of dAVF with arterial supply exclusively from the ICA, successfully treated using the Pipeline flex embolization device with Shield Technology (Medtronic, Irvine, CA, USA) [103]. Sharashidze et al. described a sigmoid sinus dAVF treated with two overlapping pipeline embolization devices (PED, Medtronic, Irvine, CA, USA) deployed across the sinus, achieving complete occlusion of the shunt while preserving sinus patency [104]. More recently, a Flow Re-Direction Endoluminal Device (FRED; MicroVention) was used to treat a craniocervical junction VA dAVF (Borden Type III), with post-treatment MR angiography confirming complete disconnection [105].

These cases suggest that flow diversion may be a viable option in select dAVFs where venous sinus preservation is critical and other treatments—such as transvenous or transarterial embolization, surgery, or radiosurgery—have failed or are not feasible. However, its utility remains limited for the majority of dAVFs due to complex angioarchitecture and the presence of multiple arterial feeders originating from different major arteries, including the ECA, ICA and VA [1].

#### 3.4.4. Transvenous Embolization

Prior to the introduction of LEAs, EVT of dAVFs relied predominantly on the transvenous route, as TAE was limited by low occlusion rates, with success achieved in only around half of cases [1]. A key factor for successful treatment is ensuring that the targeted venous structure drains exclusively the fistula, without serving as a conduit for normal cerebral venous outflow. Only by completely occluding this specific venous pathway can a durable cure be achieved [1].

TVE is indicated for dAVFs in which TAE is considered technically challenging or carries a high procedural risk. This includes cases where the dAVF is supplied by small, tortuous arteries that preclude safe distal microcatheter navigation; by vessels with high-risk extracranial-to-intracranial anastomoses, increasing the risk of parenchymal infarction; or by arteries serving as vasa nervorum, where embolization may result in ischemic cranial neuropathy [106]. These angioarchitectural challenges are particularly common in dAVFs located near the ventral skull base, such as cavernous, condylar, or clival fistulas [107]. TVE is also considered the best therapeutic approach for Cognard type II dural fistulas, with or without CVR [1].

Balloon catheters can be used both to protect the venous sinuses and non-target veins from unintended embolization and to enhance fistula penetration with LEAs. This is particularly important in dAVFs with a high risk of venous non-target embolization, especially those involving the transverse or sigmoid, sinus since such embolization can obstruct critical venous drainage pathways, potentially leading to venous congestion, infarction, or ICH [100]. Alternatively, the technically more demanding reverse pressure cooker technique (RPCT), a transvenous adaptation of the PCT, may be employed. In RPCT, a coil and/or LEA plug is formed within the draining vein, and a second microcatheter is positioned proximally, often near the foot of the draining vein. After plug formation arrests flow, embolic material is injected behind the plug, creating pressure that forces retrograde penetration of the agent toward the fistulous site [108]. However, akin to PCT, RPCT remains technically demanding and time-intensive, thereby rendering balloon-assisted techniques an appealing alternative due to their relative procedural efficiency and reduced technical complexity.

#### 3.4.5. Combined Approaches

**Figure 1:**
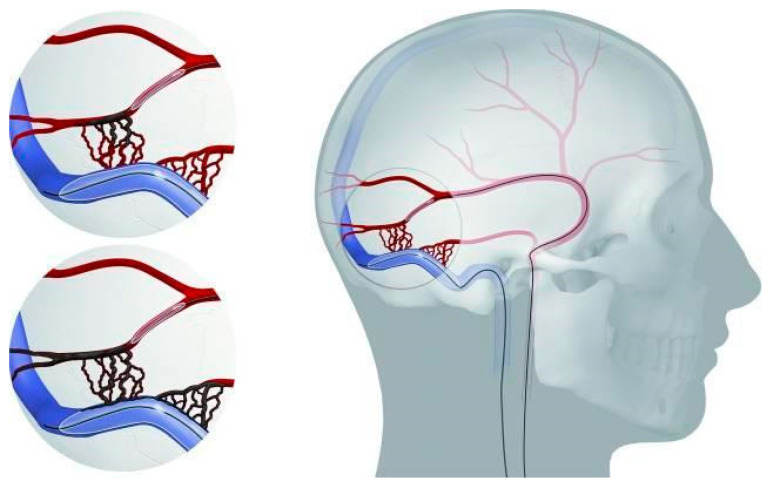
Schematic presentation of the transarterial approach (TAE) and the transvenous approach (TVE) in combined sinus-preserving embolization (CSPE) technique (Reused from Piechowiak et al.) [101].

Given the limitations associated with TAE and TVE when performed in isolation—namely, the risk of incomplete fistula occlusion in TAE and disruption of venous drainage in TVE—combined approaches have been developed to enhance efficacy while minimizing complications [Figure 1]. The combination of both TAE and TVE—by occluding the draining sinus compartment from the venous side and simultaneously embolizing the arterial feeders—is referred to as combined sinus-occluding embolization (CSOE), and was developed to increase the likelihood of achieving definitive fistula occlusion. However, CSOE carries the same potential risk as standalone TVE: compromise of the physiological intracranial venous drainage pathways. Consequently, the combined sinus-preserving embolization (CSPE) technique has been proposed. In this approach, a temporarily inflated large-lumen compliant balloon is positioned within the affected sinus compartment during Onyx injection into the arterial feeder. This strategy preserves the natural venous drainage pattern while promoting deeper and more targeted penetration of the fistula network by the liquid embolic agent [88].

A retrospective comparison of both techniques found that the CSOE approaches offered a higher rate of definitive fistula occlusion (93% vs. 71% CSPE) but were accompanied by a significantly higher complication rate (33% vs. 0%) [88]. Similarly, Vollherbst et al. reported that CSPE achieved complete occlusion in 86.4% of cases, with an overall complication rate of 20%. Transient morbidity occurred in 8% of patients, while permanent morbidity and mortality were both 0% [100]. Based on these data, most neurointerventional centers favor CSPE as the initial therapeutic approach whenever feasible. As long as the procedure results in a functional downgrading of the fistula to a Cognard Type IIa or lower—thus effectively eliminating the risk of ICH—leaving a small residual shunt is considered an acceptable outcome. In this context, the priority is to avoid the potentially serious complications associated with sinus occlusion. Accordingly, sinus-occluding techniques should be reserved for cases in which sinus preservation fails to sufficiently reduce the fistula’s angiographic grade and hemorrhagic risk [88,100,101].

### 3.5. Location-Specific Considerations in Endovascular Management

#### 3.5.1. Transverse Sigmoid Sinus Dural Arteriovenous Fistulas

EVT has become the first-line modality for TSS-dAVFs, with reported rates of complete angiographic cure ranging from 70% to 88% depending on the embolization technique and the complexity of the vascular anatomy. In lower-grade dAVFs, TAE via the MMA is the first-line approach in many centers. Despite the MMA often being a non-dominant feeder, it offers the most straightforward route for positioning the microcatheter near the fistulous point(s). To protect the patency of the transverse/sigmoid sinus, a long compliant balloon may be introduced from the venous side. This balloon should be gently inflated with appropriate pressure, allowing effective filling of the shunting pouches (SPs) with liquid embolic agents while minimizing leakage into the sinus lumen [48,109,110].

Particular care must also be taken to preserve the patency of the vein of Labbé during embolization, as its thrombosis can lead to serious complications. Some authors recommend protecting this vein with a small, separate balloon, typically introduced from the contralateral side.

During TAE, it is critical to recognize that arterial feeders to TSS-dAVFs—including the petrosal branch of the MMA, the hypoglossal and jugular branches of the APhA, and the stylomastoid branches of the OA and PAA. They not only have anastomoses with the ICA, VA, and cerebellar arteries, but also supply cranial nerves, significantly increasing the risk of serious complications [49,111,112].

Moreover, in some cases, due to robust, tortuous, or transosseous feeders, achieving complete occlusion of the fistula with TAE alone may be difficult, necessitating alternative or adjunctive treatment strategies. In a series of 33 patients with TSS-dAVFs treated with TAE alone, complete clinical cure was achieved in only 46%, with partial symptom relief in 36%. As a result, a combined or staged endovascular approach is often required [47].

Selective transvenous embolization of the shunted pouch is an alternative effective technique for treating dAVFs without CVR (Cognard Types I and IIa), allowing for fistula occlusion while preserving normal sinus drainage. In cases where the affected sigmoid sinus is already occluded and collateral cerebral venous drainage is robust, curative embolization can be achieved via a transvenous approach with coil packing and adjunctive use of liquid embolic agents, offering high rates of durable fistula obliteration. In patients with contralateral venous sinus hypoplasia who demonstrate intolerance to balloon occlusion testing (BOT) of the ipsilateral sinus, sacrifice of the diseased venous sinus carries a substantial risk of compromising cerebral venous drainage, potentially leading to serious complications. In such cases, preservation of the affected sinus and maintenance of adequate venous outflow become paramount. Emerging therapeutic strategies, including transvenous stent deployment and balloon-assisted transarterial embolization, have increasingly been recognized as viable alternatives that allow treatment of the fistula while safeguarding sinus patency and cerebral venous drainage.

In cases with CVR (Types IIb and IIa+b), initial selective transvenous occlusion of the shunted pouch can reduce arteriovenous shunt flow prior to sinus packing, which can be definitive treatment. This stepwise approach may enhance both the safety and efficacy of transvenous treatment compared to sinus packing alone. However, in patients with contralateral sinus hypoplasia or in whom balloon occlusion testing demonstrates intolerance of ipsilateral sinus sacrifice, preservation of venous outflow becomes critical. In these cases, transvenous stent reconstruction may be considered or abovementioned balloon-assisted transarterial embolization [113].

#### 3.5.2. Superior Sagittal Sinus Dural Arteriovenous Fistulas

EVT has become the mainstay in the management of SSS-dAVFs, with reported rates of complete occlusion up to 86.3% [38]. In parasagittal dAVFs, treatment generally targets the proximal portion of the arterialized draining vein, whereas true SSS-dAVFs require direct obliteration of the arteriovenous shunt within the sinus wall.

When the SSS is not involved in fistulous drainage, TAE via the MMA alone may be sufficient. If the SSS exhibits significant stenosis, its functional contribution should be assessed using BOT. A dysfunctional sinus demonstrated by BOT may be safely occluded via TVE coil packing. Conversely, if the SSS contributes to cortical venous drainage during BOT, sinus patency must be preserved. In these cases, the fistula can be occluded via TAE while protecting the sinus using temporary TVE balloon-assisted sinus protection. Balloon protection is generally unnecessary in parasagittal fistulas with occluded or stenosed sinus segments.

The MMA represents the preferred arterial access route for SSS-region DAVFs as it provides a long reflux segment to facilitate embolization. When superselective catheterization of the MMA is not feasible due to a small caliber, scalp arteries may be used. In such cases, Onyx injection may fail to reach the fistula and may occlude only the scalp artery. To overcome this limitation, the PCT or balloon-assisted embolisation can enhance embolic penetration [51].

In the SSS-region DAVFs associated with a SP resembling a direct fistula with a large-caliber arteriovenous connection, selective catheterization of the SP may allow for coil embolization. In rare instances where a feeding artery connects directly to the SSS, a microcatheter may be navigated into the sinus to permit selective sinus packing [51].

High-flow SSS-dAVFs present additional technical challenges, as conventional TAE carries the risk of unintended migration of embolic agents into the SSS or cortical veins [51,113,114]. In these cases, flow reduction via TVE prior to TAE improves procedural safety. For SSS-dAVFs with sinus stenosis but partial preservation of physiological venous drainage, combined TAE and SSS reconstruction with stent placement can alleviate venous congestion while facilitating fistula closure. A staged approach is advised by some authors, with stent placement performed first—particularly in patients presenting with ICH or severe venous hypertension—followed by TAE to achieve durable occlusion [113].

#### 3.5.3. Cavernous Sinus Dural Arteriovenous Fistulas

The primary therapeutic objective of CS-dAVFs is to decompress the cavernous sinus by occluding the arteriovenous shunts, thereby reducing venous pressure and preventing retrograde CVR, a known risk factor for ICH and neurologic deterioration [54].

While TAE may be attempted, it is often technically challenging due to the diffuse and bilateral nature of arterial supply, which frequently arises from small dural branches of both the ICA and ECA [56]. As a result, TVE has become the preferred treatment strategy, most commonly performed via the IPS through the internal jugular vein. TVE has been associated with fewer complications than TAE, and coils are generally considered safer than liquid embolic agents [53,54,56].

A recent meta-analysis found that TVE was used in 753 of 1192 cases (63.2%), and coils were employed in 712 of 1066 procedures (57.8%). Across these studies, 85% of patients (95% CI: 69.5–96.1%) experienced complete symptom resolution, while complications occurred in 7.75% (95% CI: 3.82–12.7%), and permanent neurological deficits were rare (0.15%). The reported mortality rate was 0.1% (1 of 1043 patients) [54].

Although targeted embolization of the precise shunting point is ideal, it is often technically infeasible due to the complex venous anatomy of the cavernous sinus, limited microcatheter access, or the presence of diffuse shunting that obscures the exact fistulous site. In such scenarios, non-targeted embolization of the entire affected sinus is frequently performed. While effective, this approach carries a greater risk of cranial nerve injury or disruption of physiological venous drainage.

In a retrospective study of 198 patients, Park et al. reported targeted embolization in 47.5% of cases and non-targeted embolization in 37.9%, with the remainder receiving palliative therapy. The complication rate was significantly lower in the targeted group (2.1%) compared to the non-targeted group (9.3%). Similarly, the rate of immediate angiographic success was higher in the targeted group (93.6%) than in the non-targeted group (74.6%). However, successful completion of targeted embolization was achieved in only 55.6% of cases, highlighting the technical limitations associated with diffuse or anatomically complex fistulas [115].

In cases where the IPS is inaccessible or thrombosed, the SOV provides a well-established alternative route. The SOV may be endovascularly accessed through its anterior connections with the angular and facial veins, although this route is highly dependent on the patency and caliber of the facial venous system, which varies significantly between individuals [116]. When transfacial access is not feasible, direct surgical exposure and cannulation of the SOV remains a reliable, though more invasive, alternative for achieving endovascular access to the cavernous sinus [117].

In bilateral CS-dAVFs, TVE via the IPS is the preferred route, followed by the transfacial vein or SOV. When the TVE route is occluded, TAE via the AphA can be used [53,118,119]

EVT of bilateral CS-dAVFs may be performed in a single session or as a staged procedure. Staged embolization allows the total coil volume per session to be reduced, which may decrease the risk of postoperative cranial nerve VI palsy. However, navigating a microcatheter into the venous pouch or through connections of the cavernous sinus is more difficult in the presence of previously placed coils or Onyx. Moreover, assessing the timing of subsequent interventions can be ambiguous, and evaluating clinical outcomes or paradoxical cranial nerve VI palsy can be challenging [53,118]. While some neurointerventional centers, including ours, prefer a staged approach, comparative studies are needed to determine whether single- or multi-stage EVT is safer and more effective for bilateral CS-dAVFs.

#### 3.5.4. Parasellar Dural Arteriovenous Fistulas

Parasellar dAVFs can be effectively treated with EVT, but in many cases, the approach is limited by the absence of accessible transarterial or transvenous routes. Because not all lesions are amenable to EVT, microsurgical strategies—including coagulation of arterial feeders, disconnection of venous drainage near the fistula, or lesion resection—remain important alternatives or adjuncts.

TAE is most commonly performed via the MMA or AMA, and in rare cases through the artery of the foramen rotundum. Embolization through the MMA or AMA carries risks related to dangerous anastomoses: reflux into the ophthalmic artery branches can cause permanent blindness, while reflux into cavernous or petrosal branches may injure the trigeminal or facial nerves [120]. Similarly, reflux toward the foramen spinosum must be avoided. Because retrograde reflux is frequently encountered and high-flow shunts may limit embolic penetration, the pressure cooker technique or balloon-assisted embolization is recommended. Coiling of uninvolved MMA branches can reduce the risk of embolic migration into dangerous anastomoses, and in selected cases, temporary balloon protection within the cavernous ICA can further safeguard against reflux.

TVE is particularly useful for parasellar dAVFs supplied by multiple small feeders or by tortuous arterial routes. Potential venous access pathways include the ophthalmic and facial veins, inferior petrosal sinus and cavernous sinus, superior sagittal sinus and cortical bridging veins, the pterygoid plexus, and the deep venous system (vein of Galen and vein of Rosenthal). However, TVE should be reserved for highly selected cases, given the technical challenges of navigating these venous routes and their associated risks.

#### 3.5.5. Tentorial Dural Arteriovenous Fistulas

Because T-dAVFs are typically associated with retrograde leptomeningeal drainage, they represent aggressive lesions that require early and definitive treatment. While microsurgical disconnection of the venous outflow or coagulation of the fistula was traditionally considered the gold standard, surgery is now largely reserved for lesions with complex arterial supply, such as those arising from the MHT, which poses significant challenges for endovascular access. Increasingly, T-dAVFs are managed with EVT, which has been associated with superior neurological outcomes, albeit with lower complete occlusion rates [121].

TVE is often the preferred approach, particularly when the affected sinus is non-functional. This typically involves accessing the fistula via the SPS, with Kirsch et al. reporting an 86% cure rate using TVE with coils [47]. However, many T-dAVFs drain exclusively into subarachnoid veins (Borden type III), precluding a transvenous route. In such cases, TAE via the posterior branch of the MMA usually provides good access to the venous collector system of the fistula. The MMA’s relatively straight course and firm dural anchoring make microcatheter retrieval reliable, even in the setting of substantial Onyx reflux [121]. Nevertheless, reflux should not extend to the level of the foramen spinosum to avoid compromising arterial supply to the trigeminal and facial nerves.

A subtype of T-dAVFs, petrous dAVFs are high-grade lesions (Borden type III, Cognard types III–IV) owing to their retrograde drainage into the petrosal vein and its tributaries [122]. They may be associated with particularly aggressive neurological behavior, including subarachnoid hemorrhage (SAH), severe brainstem edema, and a high mortality rate [122].

These lesions are intimately related to the arterial arcade of the facial nerve—an anastomotic network within the petrous temporal bone that supplies the geniculate ganglion and tympanic segment of the facial nerve. The arcade is primarily formed by the petrous branch of the MMA and the stylomastoid branch of either the PAA or OA, which anastomose near the geniculate ganglion, creating a vulnerable vascular territory. Additional contribution may come from the inferior tympanic artery, a branch of the APhA, which accompanies Jacobson’s nerve into the tympanic cavity [123]. Given this intricate anatomy, embolization via ECA feeders must be performed with extreme caution to avoid iatrogenic facial nerve palsy.

TAE is generally favored when accessible non-arcade feeders supply the fistula, when a direct arteriovenous connection is present without an intervening nidus, and when a sufficient safety margin exists—that is, the microcatheter tip can be positioned at least 20 mm distal to the origin or anastomotic points of the facial nerve arterial arcade to allow reflux of embolic material without compromising its supply [123]. In the absence of these conditions, TVE may be considered if a draining vein can be safely accessed and occluded. To promote retrograde penetration of embolic agents toward the fistula, techniques such as RPCT or the use of dual-lumen balloons may be employed. Given the need to traverse delicate pial veins, these approaches carry a significant risk of venous rupture and hemorrhage.

When EVT is not feasible or fails, microsurgical disconnection of the superior petrosal vein may be pursued. Careful assessment of cerebellar venous drainage on digital subtraction angiography (DSA) is essential, as sacrifice of the vein carries a risk of cerebellar venous infarction if it represents a dominant drainage pathway.

#### 3.5.6. Anterior Cranial Fossa Dural Arteriovenous Fistulas

ACF-dAVFs are high-grade lesions, characterized by retrograde leptomeningeal venous drainage and arterial supply from the ethmoidal branches of the ophthalmic artery. Microsurgical disconnection through a tailored craniotomy allows direct exposure and interruption of the draining vein at the fistulous point and remains a highly effective strategy. In current practice, however, surgery is generally reserved for cases where EVT is unsuccessful or incomplete, reported in ~2% of failed attempts and 10% of partial occlusions in a recent meta-analysis of 231 cases. In the same analysis, complete occlusion was achieved in 85% of patients undergoing embolization, with complications in 4%, incomplete occlusion in 10%, and failure in 2%. Symptom resolution or improvement was documented in 94% of successfully embolized patients, with procedure-related morbidity of 1% and a single treatment-related death [124].

EVT can be performed with TAE, TVE, or a combined approach, with the choice depending on the specific angioarchitecture, as both approaches appear equivalent in terms of occlusion rates and complication profiles.

Among arterial routes, the ophthalmic artery is generally preferred over the MMA or sphenopalatine arteries because its higher flow facilitates deeper penetration of a LEA toward the fistulous point and draining vein, thereby improving the likelihood of durable occlusion [125]. Catheterization, however, may be challenging as the ophthalmic artery commonly arises at an acute angle from the ICA. In such scenarios, temporary ICA balloon occlusion can facilitate microcatheter navigation. To minimize the risk of retinal ischemia, the microcatheter should be advanced distal to the origin of the central retinal artery [125,126]. A diagnostic angiogram from the guiding catheter is then essential to confirm the microcatheter position relative to the central retinal artery, assess ophthalmic artery patency, and exclude flow-limiting vasospasm; if vasospasm is encountered, intra-arterial vasodilators are recommended to maintain retinal perfusion [126].

Because the margin for reflux is limited in this vascular territory, placement of a microballoon distal to the central retinal artery and as close to the fistulous point as possible can enhance safety and embolization efficacy [127]. PCT at this site is technically demanding and is generally discouraged. Importantly, despite theoretical risk, no cases of permanent blindness have been reported to date.

When transarterial access is not feasible, retrograde embolization via the SSS and frontobasal vein offers an alternative [126]. This strategy avoids ophthalmic artery catheterization and the associated risk of retinal ischemia, but requires negotiation of tortuous venous anatomy, with feasibility determined by individual venous drainage patterns.

Despite these evolving strategies, the optimal management of ACF-dAVFs remains a subject of ongoing debate. In the CONDOR registry, which prospectively captured outcomes of patients with ACF-dAVFs, microsurgical disconnection was associated with a complete occlusion rate of 100% (35/35), compared to 53% (9/17) in patients treated with EVT. Additionally, microsurgery carried a lower complication rate (6%, 2/35) versus EVT (12%, 2/17) [69]. These findings are echoed in a meta-analysis of 20 studies comprising 224 patients, which reported significantly higher complete obliteration rates for microsurgery than EVT (89% vs. 70%, respectively) [128]. Conversely, more recent single-center series underscore the potential of EVT in experienced hands. Su et al. employed TAE as the first-line approach in 40 patients, achieving an immediate angiographic cure rate of 82.5%, with only one reported complication and no recurrences [129]. Likewise, Trivelato et al. reported on 35 patients treated primarily with EVT, of whom 19 underwent exclusive TAE, resulting in complete occlusion in 84.2% (16/19) and a complication rate of 10.5% (2/19) [126]. In view of these data, the choice of treatment should be individualized, taking into account the patient’s clinical presentation, lesion angioarchitecture, and the expertise of the treating team.

#### 3.5.7. Anterior Condylar Confluence Dural Arteriovenous Fistula

EVT represents the primary treatment for ACC-dAVFs, with an angiographic cure rate of 93.4%, permanent morbidity of 5.4%, and no reported mortality. Although microsurgical disconnection of dAVFs can be both safe and effective, lesions at the skull base carry a higher operative risk profile than those at other locations, owing to the intraosseous position of the shunt, proximity to lower cranial nerves, and the potential need for condylar bone resection, which may result in craniocervical instability [130,131]. Accordingly, microsurgery is generally reserved for refractory cases or when EVT is not feasible.

TVE achieves the highest clinical cure rate (91%) with relatively low morbidity (2.9%), most commonly via the ipsilateral IJV. When direct access is precluded by hypoplasia, tortuosity, or occlusion of the IPS, alternative routes through the contralateral IJV, contralateral IPS, intercavernous sinus, or ipsilateral cavernous sinus can be exploited [70,130]. The SOV may also provide access, either via the facial vein, percutaneous puncture, or surgical cutdown, although the latter carries the risk of injury to the superior root of the trigeminal nerve [130].

Coils remain the most widely used embolic material. Although LEAs can effectively diminish shunt flow, they are rarely curative as a standalone strategy. To reduce the risk of hypoglossal nerve palsy from coil mass effect within the ACV, the use of soft coils and avoidance of excessive or overly compact packing is recommended [70].

TAE may be considered for type III ACC-dAVFs with exclusive cortical or perimedullary venous drainage, or in cases where TVE is not feasible. However, arterial supply most often arises from the APhA, OA, and meningeal branches of the VA, which maintain dangerous anastomotic connections with the brainstem feeders, rendering primary TAE high-risk [17,33]. In addition, arterial feeders are frequently small and tortuous, complicating stable distal catheterization. The neuromeningeal division of the APhA also provides supply to cranial nerves IX–XII, creating a recognized risk of lower cranial neuropathy following embolization.

### 3.6. Surgery

While EVT is the preferred treatment for low-grade dAVFs, with recent CONDOR data showing that complete angiographic occlusion provides longer symptom-free survival compared to partially embolized or untreated fistulas [89], the optimal approach for higher-grade dAVFs is more nuanced, highlighting the need for a tailored, risk-adapted strategy. The CONDOR registry reported that microsurgery achieved higher obliteration rates for Borden 2 and 3 dAVFs (86%) compared to EVT (43.3%) and SRS (30.8%). However, hemorrhage rates per 1000 patient-years were significantly lower following EVT (9, *p* = 0.022) than surgery (22, *p* = 0.245), with SRS showing no hemorrhages (0, *p* = 0.077) [131]. These findings support embolization as the first-line treatment for appropriately selected unruptured Borden type II and III dAVFs due to its association with a reduced risk of hemorrhage and death [131]. Nonetheless, surgery remains an effective and safe—albeit more invasive—option for higher-grade dAVFs when endovascular options carry high risk, are not feasible, or have been exhausted [132]. Moreover, certain dAVF locations are inherently less amenable to EVT and can be effectively managed with a few tailored surgical approaches that carry acceptable rates of major complications [132]. Notably, analysis of 248 patients from the CONDOR database demonstrated that microsurgery, with or without adjunctive embolization, achieved significantly higher rates of angiographic obliteration or downgrading than embolization alone for tentorial, anterior cranial fossa, and torcular fistulas, without increasing permanent neurological morbidity [132]. Likewise, in convexity and SSS-dAVFs, microsurgery represents an attractive alternative to EVT when tortuous arterial feeders prevent distal catheterization or when transvenous routes are unavailable due to venous sinus occlusion. Conversely, in TSS dAVFs, although surgery offers higher rates of angiographic obliteration or downgrading compared to EVT (93.1% vs. 62.7%), it carries a significantly greater risk of permanent neurological deficit (9.1% vs. 2.3%) [132]. Therefore, despite lower efficacy, EVT often remains the safer choice for TSS dAVFs. Ultimately, the choice between EVT and microsurgery should be guided by patient-specific risks and benefits, as well as the treating team’s expertise with endovascular and surgical techniques.

### 3.7. Stereotactic Radiosurgery

Although SRS is infrequently used as sole therapy for dAVFs—with only 36 of 1077 patients in the CONDOR database treated exclusively with SRS—it remains an effective and minimally invasive option [91]. Pooled data indicate a CO rate of approximately 70%, alongside symptom improvement or cure reported in about 97% and 80% of patients, respectively. Importantly, rates of permanent neurological deficits and hemorrhage after SRS are low, around 1%; however, patients with a history of prior hemorrhage have an increased risk of hemorrhage following SRS [19].

Several factors influence SRS outcomes. Studies consistently report higher CO rates in CS dAVFs compared to non-CS locations [5,133]. Prior embolization, by reducing fistula size and facilitating safer SRS delivery, is associated with improved symptom cure rates, although it carries the risk of obscuring the dAVF on stereotactic imaging [134]. While some research suggests that CVD negatively impacts CO rates [135], other studies find no significant effect [136]. Additionally, venous ectasia and high-flow shunting have been linked to incomplete obliteration [136]. Presentation with NHNDs is also a risk factor for developing new permanent neurological deficits after SRS [134].

Based on current evidence, the International Stereotactic Radiosurgery Society Practice Guidelines recommend SRS for: patients with complex dAVFs planned for embolization who are at high risk of failing to achieve CO with embolization alone, and patients with dAVFs who have undergone prior embolization without CO and have refractory symptoms—particularly when microsurgery is not feasible or is declined [19].

Given the high hemorrhage risk in unruptured high-grade dAVFs and the recurrent hemorrhage risk in ruptured dAVFs, SRS is generally considered suboptimal due to its relatively prolonged latency period before obliteration, with reported angiographic cure rates around 50% [3,135,137]. Nevertheless, for patients who are not candidates for or refuse both embolization and microsurgery, SRS remains a viable option, offering potential angiographic cure [134] and symptom relief with low rates of adverse events [136].

Management of Borden type I dAVFs is controversial, as the relative benefits of observation versus intervention remain uncertain. A recent meta-analysis demonstrated that intervention in Borden type I dAVFs was associated with a higher risk of death and permanent complications compared with observation (3.9% vs. 0%; risk difference = 0.04 [0.01–0.06]) [138]. Observation appears most appropriate for asymptomatic patients, whereas intervention is justified in those with limiting symptoms to enhance quality of life. Although EVT is the most frequently employed approach, certain anatomical and angiographic features can limit its efficacy, often resulting in partial rather than complete embolization [139]. This, combined with the risk of complications, has increased interest in SRS as an alternative. SRS offers favorable obliteration rates with relatively low treatment-related morbidity. In addition to promoting long-term cure, studies suggest SRS may reduce perilesional inflammation, thereby facilitating earlier symptom improvement [140]. A recent study reported that single-fraction SRS achieved higher rates of symptomatic improvement (95.1% vs. 58.5%; OR 13.75, 95% CI 5.61–33.69) and mRS improvement (37.0% vs. 24.0%; OR 1.85, 95% CI 1.09–3.15) compared with observation [139]. SRS was associated with adverse events in just 3% of patients and no permanent morbidity, in stark contrast to EVT, which carries a 5–22% complication rate [141,142,143,144,145,146].

Taken together, these findings highlight SRS as a safe and effective treatment option for symptomatic low-grade dAVFs. Nonetheless, the current evidence is largely derived from retrospective series and non-randomized comparisons. Further prospective, multicenter studies are warranted to better define the optimal role of SRS in the management algorithm of low-grade dAVFs.

## 4. Conclusions

DAVFs are complex vascular lesions defined by significant anatomical and hemodynamic heterogeneity. Although outcomes for dAVFs have improved thanks to a better understanding of their pathology and the availability of newer treatment modalities, their multifactorial pathogenesis remains incompletely understood, underscoring the need for further research into underlying mechanisms and risk factors.

Although various classification systems exist to estimate the risk of hemorrhage or ischemic neurological injury in dAVFs, the presence of CVD and venous ectasia is the strongest predictor of adverse neurological outcomes and should be the basis for making treatment decisions.

Optimal management of intracranial dAVFs depends on meticulous angiographic assessment and multidisciplinary collaboration to tailor therapy according to lesion anatomy and clinical context. Patients with CVD and aggressive clinical manifestations fall into the highest-risk category and require urgent intervention, with the primary aim of disconnecting the venous drainage and, when feasible, achieving complete obliteration of the fistula. Patients with CVD but without aggressive symptoms or venous ectasia face a comparatively lower yet still clinically relevant risk, warranting prompt treatment. In contrast, dAVFs lacking CVD typically follow a benign course and are treated only if they cause significant symptoms such as intolerable tinnitus or ocular manifestations, with symptom relief rather than complete obliteration being the goal. Patients managed conservatively should undergo close clinical surveillance, as any change in symptomatology, including improvement, warrants prompt angiographic evaluation.

Low-profile microcatheters, high-torque guidewires, liquid embolic agents, dual-lumen balloons, and high-definition angiography have made the EVT a highly feasible, effective, and safe method, with a low rate of complications and low procedure-related morbidity and mortality. Continuing technical advances, such as steerable microguidewires, are bound to improve vessel navigability, reduce fluoroscopy times, and further optimize this approach. When possible, sinus-preserving strategies are preferred to maintain normal venous drainage and minimize complications, while sinus-occluding techniques are reserved for cases where preservation does not sufficiently reduce the hemorrhagic risk. Microsurgery remains a valuable alternative, especially for ACF- and T-dAVFs, which show high obliteration rates after surgery. Radiosurgery provides a minimally invasive option for patients with complex dAVFs, particularly when embolization is predicted to be insufficient or prior attempts have failed, and for high-risk patients who are not candidates for, or decline, embolization or microsurgery. Although retrospective studies suggest favorable outcomes for symptomatic patients with low-grade lesions, prospective, multicenter studies are needed to define its precise role. Given that selection criteria for radiosurgery remain incompletely established, with factors such as CVD, prior ICH, and fistula location influencing clinical decisions, further research should clarify which patients are most likely to benefit from SRS.

Limitations: While this review provides a broad and detailed synthesis of available literature, it does not allow for definitive conclusions regarding the comparative safety or efficacy of specific treatment modalities. The lack of quantitative synthesis further limits our ability to evaluate outcomes in a statistically meaningful way. Moreover, the included studies exhibited considerable heterogeneity in terms of design, patient populations, anatomical DAVF subtypes, and outcome measures. This variability complicates direct comparisons and generalizability, particularly given the rarity and complex angioarchitectural spectrum of DAVFs. Additionally, much of the data stems from retrospective, single-center experiences conducted in high-volume tertiary institutions, which may not be generalizable to smaller centers with limited endovascular resources or experience.

## Figures and Tables

**Table 1:** Vascular Supply and Drainage of dAVFs by Location.

Anatomical Location		Main Feeders	Main Drainers
CS-dAVF		ECA/ICA branches, mainly MMA, accessory meningeal artery, APhA	CS, SOV, IPS
TSS-dAVF		MMA, PMA, and meningeal branches of the APhA and OA	TSS, SSS, superior petrosal sinus, basal vein of Rosenthal, vein of Labbé, cortical and deep medullary veins
SSS-dAVF		MMA, superficial temporal artery, occipital artery, posterior meningeal artery, pial supply from the cerebral arteries (rarely)	SSS, bilateral superficial CV, vein of Labbé
T-dAVF		artery of Bernasconi and Cassinari, petrosal and petrosquamous branches of the MMA, meningeal branch of the OA, PMA,	through the pontine, perimesencephalic, and the basal veins into the Galenic system
ACF/Ethmoidal-dAVF		anterior ethmoidal artery, MMA, ethmoidal branches of IMA	To the frontal veins and then secondarily into the SSS, via the olfactory vein to the CS or the basal vein of Rosenthal, or to the Sylvian veins and then ultimately into the vein of Trolard or Labbé
Parasellar region (groups)	Anterior	ophthalmic artery	Ophthalmic vein
	Anterolateral	ECA, ILT	Sphenoparietal sinus
	Posteroinferior	ECA, APhA, MHT	IPS, Basilar venous plexus
	Posterior	MMA, MHT	Petrosal vein
ACC-dAVF		APhA (most common), ophthalmic artery, MMA, PAA, MHT, VA	IJV, VVP, IPS, CS, MS

ACF: anterior cranial fossa; ACC—anterior condylar confluence; APhA: ascending pharyngeal artery; CS: cavernous sinus; CS-dAVF: cavernous sinus dural arteriovenous fistula; CV: cortical veins; dAVF: dural arteriovenous fistula; ECA: external carotid artery; ICA: internal carotid artery; IJV: internal jugular vein; ILT: inferolateral trunk; IMA: internal maxillary artery; IPS: inferior petrosal sinus; MHT: meningohypophyseal trunk; MMA: middle meningeal artery; MS: marginal sinus; OA: occipital artery; PAA: posterior auricular artery; PMA: posterior meningeal artery; SOV: superior ophthalmic vein; SSS: superior sagittal sinus; T-dAVF: tentorial dural arteriovenous fistula; TSS: transverse-sigmoid sinus; TSS-dAVF: transverse-sigmoid sinus dural arteriovenous fistula; VA: vertebral artery; VVP: vertebral venous plexus.

**Table 2:** Characteristics and symptoms of CS- and Parasellar dAVFs (P-dAVFs) (reused from Hiramatsu et al.) [36].

Group	Location	Main Feeder	Main Drainer	Symptom
CS	Cavernous sinus (CS)	ECA, ILT, MHT	CS SOV/IPS	Benign
Anterior	Orbit	Ophthalmic artery	Ophthalmic veins	Aggressive
Anterolateral	Lesser sphenoid wing	ECA, ILT	Sphenoparietal sinus	Benign
	Greater sphenoid wing	ECA, ILT	SMCV	Aggressive
Posteroinferior	Inferior petrosal sinus	ECA	IPS	Benign
	Clivus (osseous/dura)	APhA, MHT	Basilar venous plexus	Benign
Posterior	Superior petrosal sinus	MMA, MHT	Petrosal vein	Aggressive

APhA: ascending pharyngeal artery; AVF: arteriovenous fistula; CS: cavernous sinus; ECA: external carotid artery; ILT: inferolateral trunk; IPS: inferior petrosal sinus; MHT: meningohypophyseal trunk; MMA: middle meningeal artery; SMCV: superficial middle cerebral vein; SOV: superior ophthalmic vein.

**Table 3:** Lawton–Halbach classification of tentorial dAVFs [60].

dAVF	Location	Dural Base	Venous Sinus	Venous Drainage
Galenic	Midline	Anterior falcotentorial junction	Vein of Galen	Supra- and infratentorial
Straight sinus	Midline	Middle falcotentorial	Straight sinus	Infratentorial
Torcular	Midline	Posterior falcotentoria	Torcula	Supratentoria
Tentorial sinus	Paramedian	Tentorium	Tentorial sinus	Supratentoria
Superior petrosal sinus	Lateral	Petrotentorial junction	Superior petrosal sinus	Infratentorial
Incisural	Paramedian	Tentorial incisura	None	Supratentorial

**Table 4:** Groups of T-dAVF according to Picard et al. [61].

Group	Location	Venous Drainage	Drainage Pattern
Medial Tentorial Sinus Group	Adjacent to the torcular	Cerebellar hemispheres and vermis (infratentorial drainage)	Drainage into the torcular, lateral sinus, or straight sinus
Lateral Tentorial Sinus Group	Adjacent to the lateral sinus	Lateral and inferior surfaces of the temporal and occipital lobes (supratentorial drainage)	Drainage into the lateral sinus
Marginal Tentorial Sinus Group	Along the free edge of the tentorium	Basilar and lateral mesencephalic veins	Infra- or supratentorial drainage/drainage into spinal veins

**Table 5:** Groups of T-dAVF according to Lasjaunias et al. [62].

Group (Region)	Venous Drainage
Torcular	Medial occipital and infratemporal areas
Basal Tentorium	Superior petrosal sinus and petrosal vein
Marginal Tentorium	Tentorial vein, vein of Rosenthal, and mesencephalic veins

**Table 7:** Comparison of LEAs.

Agent	Advantages	Disadvantages
n-BCA	Rapid polymerization enables quick, durable vessel occlusion; historically most used LEA	Operator dependent; requires wedged microcatheter position; off-label use in many regions
Onyx	Proven efficacy; widespread use; less operator dependent; does not require wedged microcatheter position; slower, controlled delivery than n-BCA	Requires preparation time; more pronounced risk of reflux compared to n-BCA
Squid	Lower tantalum content (30%) with micronized particles improves visibility and homogeneity versus Onyx; available as Squid 18 (higher density for plug formation) and Squid 12 (lower viscosity for distal penetration)	Newer agent; limited long-term data; not commercially available in the USA; more pronounced risk of reflux compared to n-BCA; requires preparation time
PHIL	No preparation needed; fewer CT artifacts; low viscosity	Newer agent; limited long-term data; not commercially available in the USA ^; more pronounced risk of reflux compared to n-BCA

LEA—liquid embolic agent; n-BCA—n-butyl-2-cyanoacrylate, CT—computed tomography, ^—The U.S. FDA granted Humanitarian Device Exemption (HDE) approval for the PHIL Embolic System in June 2016 for the treatment of intracranial dural arteriovenous fistulas (dAVFs).
